# The Association of 25-Hydroxyvitamin D_3_ and D_2_ with Behavioural Problems in Childhood

**DOI:** 10.1371/journal.pone.0040097

**Published:** 2012-07-10

**Authors:** Anna-Maija Tolppanen, Adrian Sayers, William D. Fraser, Glyn Lewis, Stanley Zammit, Debbie A. Lawlor

**Affiliations:** 1 Medical Research Council Centre for Causal Analyses in Translational Epidemiology, School of Social and Community Medicine, University of Bristol, Bristol, United Kingdom; 2 School of Social and Community Medicine, University of Bristol, Bristol, United Kingdom; 3 Norwich Medical School, University of East Anglia, Norwich, United Kingdom; 4 Medical Research Council Centre Centre for Neuropsychiatric Genetics and Genomics, Cardiff University, Cardiff, United Kingdom; Federal University of Rio de Janeiro, Brazil

## Abstract

**Background:**

Higher serum concentrations of 25-hydroxyvitamin D (25(OH)D), an indicator of vitamin D synthesis and intake, have been associated with better mental health and cognitive function. Concentrations of 1,25-dihydroxyvitamin D_3_ (the active vitamin D_3_ metabolite) have been associated with openness and extrovert behaviour, but 25(OH)D concentrations have not been associated with behavioural problems in humans.

**Methods:**

We investigated the prospective association between the different forms of 25(OH)D - 25(OH)D_3_ and 25(OH)D_2_– and childhood behavioural problems in Avon Longitudinal Study of Parents and Children (ALSPAC). Serum 25(OH)D_3_ and 25(OH)D_2_ concentrations were assessed at mean age 9.9 years. Incident behavioural problems were assessed with Strengths and Difficulties Questionnaire (SDQ; emotional symptoms, conduct problems, hyperactivity-inattention problems, peer relationship problems and pro-social behaviour subscales and total difficulties score) at mean age 11.7. Sample sizes varied between 2413-2666 depending on the outcome.

**Results:**

Higher 25(OH)D_3_ concentrations were weakly associated with lower risk of prosocial problems (fully adjusted odds ratio: OR (95% confidence interval: CI) 0.85 (0.74, 0.98)). Serum 25(OH)D_3_ or 25(OH)D_2_ concentrations were not associated with other subscales of SDQ or total difficulties score after adjusting for concfounders and other measured analytes related to vitamin D.

**Conclusions:**

Our findings do not support the hypothesis that 25-hydroxyvitamin D status in childhood has important influences on behavioural traits in humans.

## Introduction

Higher serum concentrations of 25-hydroxyvitamin D (25(OH)D), an indicator of dietary intake and cutaneous synthesis of vitamin D [1], have been associated with healthier cognitive and mental health phenotypes, including better cognitive function [2]–[7], and reduced risk of schizophrenia [8], [9] and depression [10]–[12], albeit with some inconsistencies between studies [13]–[15]. Animal experiments have shown associations between vitamin D deficiency and aberrant behaviour, social functioning and lower habituation (reviewed in [16]–[18]). A small study (n = 206) found that higher serum 1,25-dihydroxyvitamin D_3_ (1,25(OH)_2_D_3_) concentrations were associated with more extrovert and open behaviour in adults [19], while maternal serum 25(OH)D concentrations during second [20] or third [21] trimester were not associated with behavioural problems in children.

Circulatory 25(OH)D consists of 25(OH)D_3_ (synthesised from vitamin D_3_ obtained mainly from skin synthesis and to a lesser extent from animal food sources) and 25(OH)D_2_ (synthesised from vitamin D_2_ obtained from certain plant food sources). 25(OH)D_3_ and 25(OH)D_2_ are converted to 1,25-dihydroxyvitamin D_3_ and D_2_, the steroid hormones that mediate the biological actions of vitamin D. The former has higher affinity to vitamin D binding protein [22], [23] and with respect to bone health, vitamin D_3_ has been suggested to be more potent than D_2_ [24]. The aims of this study were to investigate the association of serum 25(OH)D_3_ and 25(OH)D_2_ concentrations with behavioural problems in childhood and to compare whether the associations of 25(OH)D_3_ and 25(OH)D_2_ are different from each other. Because vitamin D, together with parathyroid hormone (PTH), regulates calcium and phosphate homoeostasis ^23;24^ we investigated whether the associations of 25(OH)D_3_ and 25(OH)D_2_ were independent of PTH, calcium and phosphate concentrations.

## Methods

### Population

The Avon Longitudinal Study of Parents and Children (ALSPAC) is a population-based birth cohort from South West England. The cohort consisted of 14062 live births from 14541 pregnant women who were expected to give birth between April 1, 1991, and December 31, 1992 [25]. From age 7, all children were invited for an annual assessment of physical and psychological development. Parents gave written informed consent at enrolment, and ethical approval was obtained from the ALSPAC Law and Ethics Research Committee and the National Health Service (NHS) local research ethics committee.

**Figure 1:**
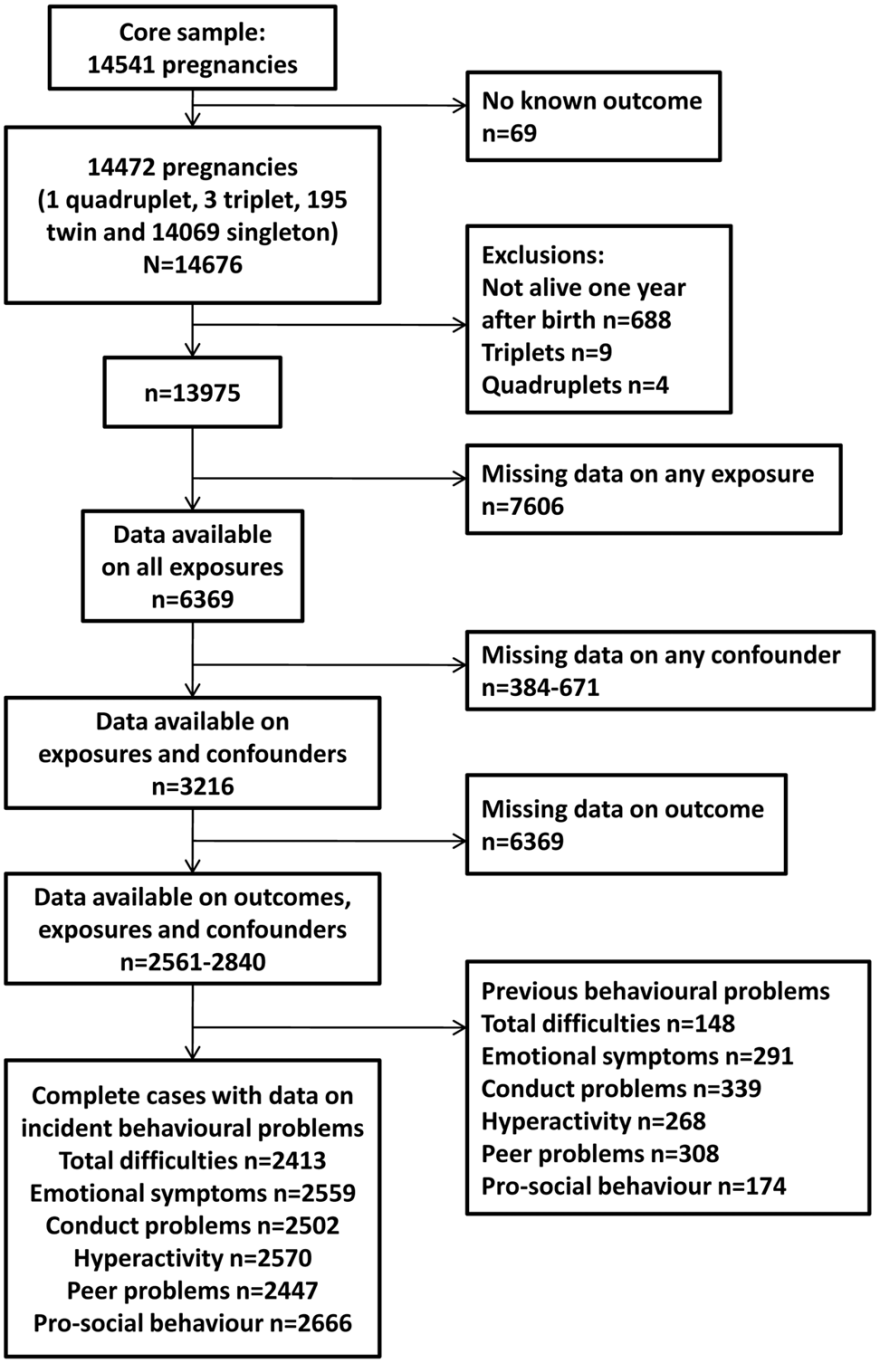
Flow of participants.

Single and twin births were included in this study; the very small number of triplets and quadruplets were not included for reasons of confidentiality. Figure 1 shows how the number included in the analyses was derived. Depending on outcome, 2413–2666 children had complete data on outcome, exposures and confounders.

### Exposures and Blood Based Covariables

Serum 25(OH)D_3_, 25(OH)D_2_, PTH, phosphate and calcium concentrations were assayed on non-fasting blood samples collected at mean age 9.9 years for the majority of participants. If no samples were available from the 9.9 years assessment, samples from mean age 7.6 years or, secondly, the 11.8 years assessment. The mean age at sample collection in the whole study sample was 9.9 years (standard deviation: SD 1.05). Following collection, samples were immediately spun, frozen and stored at –80°c. 25(OH)D_3_ and 25(OH)D_2_ assays were performed with high pressure liquid chromatography tandem mass spectrometer (Waters Acuity, Manchester, UK) after a maximum of 12 years in storage with no previous freeze-thaw cycles as described previously [26]. Inter-assay coefficients of variation for the assay were <10% across a working range of 2.5–624 nmol/L for both 25(OH)D_3_ and 25(OH)D_2_. Measurements were performed in a laboratory meeting the performance target set by the Vitamin D External Quality Assessment Scheme (DEQAS) Advisory Panel for 25(OH)D assays.

Total serum calcium, phosphate and albumin concentrations were measured by standard laboratory methods on Roche Modular analysers (Roche Diagnostics Ltd, West Sussex, UK). Serum calcium was adjusted for albumin using a normogram of calcium and albumin distributions of the samples analysed in the clinical chemistry laboratory where the measurements were performed and albumin-adjusted calcium was used in all statistical analyses. Intact parathyroid hormone (iPTH(1-84)) was measured by electrochemiluminescent immunoassay on Elecsys 2010 immunoanalyzer (Roche, Lewes, UK). Inter-assay coefficient of variation was <6% from 2–50 pmol/L. The assay sensitivity was 1 pmol/L.

### Outcomes

Behavioural problems were assessed using the Strengths and Difficulties Questionnaire (SDQ), completed by parents when the children were at mean ages 7.6, 9.6 and 11.7 years. The SDQ is a behavioural screening questionnaire that has been validated for use in children aged 4–16 years [27]. It includes 25 items relating to positive and negative behaviours and these are used to score children on five subscales: emotional symptoms, conduct problems, hyperactivity-inattention problems, peer relationship problems and pro-social behaviour. Scores on each scale range from zero to ten and higher scores denote more problems, except on the reverse-scored pro-social subscale in which higher scores indicate more social behaviour. A total difficulties score (range 0–40) is obtained by summing the scores for the emotional symptoms, conduct problems, hyperactivity-inattention problems and peer relationship problems scales. Only children whose parents answered all questions in each subscale were included in this study.

Total difficulties score and scores from five subscales were dichotomised according to cut-off values for borderline/abnormal behaviour according to parent-rated SDQ (>13 for total difficulties, >3 for emotional symptoms, >5 for hyperactivity-inattention problems, >2 for conduct and peer problems and <6 for prosocial behaviour). Full description of questionnaire and information on cut-offs and normative values are available at http://www.sdqinfo.org. In order to assess prospective associations of exposures with incident behavioural problems at age 11.7 years and to ensure that no associations were due to reverse causality, we excluded any children with behavioural problems at the age 7.6 or 9.6 assessments.

### Confounders

We considered ethnicity (white, non-white), head of household occupational social class, maternal and paternal education, family history of depression or schizophrenia, exposure to ultraviolet B (UVB), body mass index (BMI) and cognitive function to be important confounders because of their known associations with 25(OH)D_3_ concentrations and behavioural problems. We also adjusted for pubertal stage as this might affect behavioural problems and 25(OH)D_3_.

Data on head of household social class, ethnicity, maternal and paternal education and family history of depression and schizophrenia were obtained from parent-completed questionnaires. Time spent outdoors during summer months on school days, school weekends and holidays was reported as ‘None’, ‘1 hour/day’, ‘1–2 hours/day’ and ‘3 or more hours/day’ in parent-completed questionnaires at mean age of 8.5 years. Responses were coded as follows: ‘Nonè = 0, ‘1’ = 1, ‘1–2’ = 1.5 and ‘3’ = 5. Average hours spent outdoors per summer day (June 1-August 31) were calculated using term dates from Bristol City Council’s Education Committee term dates for 2001-2002 (summer term June 1-July 23, holidays July 24-August 31). Information on protection from UVB exposure (use of sunblock, covering clothing or hat and avoidance of midday sun) were obtained from the same questionnaires. A summary variable for UVB protection score was derived by scoring the responses to questions on use of sunblock, covering clothing or hat and avoidance of midday sun as ‘Always’ = 3, ‘Usually’ = 2 ‘Sometimes’ = 1 ‘Never’ = 0 and summing these scores. This gives a single variable that ranges from zero to twelve, with zero indicating the least meticulous protection from UVB.

Height and weight were measured at the same time as blood samples for obtaining 25(OH)D_3_ and other assays and were used to calculate body mass index (BMI). Total IQ score in Wechsler Intelligence Scale for Children (WISC-III UK version) was assessed at mean age 8.5. Puberty stage was assessed by parental report using Tanner staging [28] of breast, genitalia development and pubic hair on repeat occasions. In our analyses we used data from the questionnaire closest to the time of phlebotomy for the exposures for each child.

### Statistics

Statistical analyses were conducted with Stata 11.0 (Stata Corp LP, College Station, TX USA). To adjust for seasonal variability in 25(OH)D_3_, the latter was modeled according to date of blood sampling using linear regression with trigonometric sine and cosine functions. 25(OH)D_3_ was log_e_ transformed to reduce heteroscedasticity. The residual was then used as 25(OH)D_3_ exposure variable in regression analyses of the main results. In supplementary analyses we also report associations unadjusted (for season) 25(OH)D_3_ and of total 25(OH)D [25(OH)D_3_ plus 25(OH)D_2_]. To include all participants on whom a 25(OH)D_2_ was assayed, those with a value below the detectable limit of the assay (1.25 nmol/L, N = 1242-1622 depending on outcome) were indicated using a binary covariable in all regression models. In order to take account of age differences at the time of assessment we generated age and gender standard deviation scores for serum 25(OH)D_3_, 25(OH)D_2,_ calcium, phosphate and PTH using the internal cohort data with age in 1 month categories.

The association of potential confounders with exposures was assessed with linear regression and associations of confounders with SDQ scales problems with logistic regression. Other analyses were performed using a non-parametric bootstrap procedure in conjunction with logistic regression, based on 1000 replications. The bootstrapping procedure enabled us to statistically compare associations of 25(OH)D_3_ with behavioural problems to those of 25(OH)D_2_ with behavioural problems. The difference between the association of 25(OH)D_3_ and 25(OH)D_2_ was calculated from the bootstrap replicate distribution. Beta estimates and standard errors were empirically calculated from the mean and standard deviation of the bootstrap distribution respectively. All *P*-values were calculated using bootstrap means and standard errors and compared to a z-distribution, 95% percentile confidence intervals were calculated. In order to numerically compare the associations of two forms of 25(OH)D, we scaled them the same by multiplying the beta coefficients from the regression models by log_e_(2). Analyses were performed for both genders combined as there was no statistical evidence of gender*exposure interaction (all P≥0.20).

Because some children had exposure and outcome measured at the same time (14.3–15.0%, n = 350–399 depending on outcome), a separate analyses restricted to those with prospective exposure measurement (age 7.6 or 9.9 years) was done to see if the exclusion of more cross-sectional data affected the association. We also assessed the association between the exposures and SDQ scores with Poisson regression. These results are shown as supplementary material.

## Results

The median (interquartile range) serum season-adjusted 25(OH)D_3_, 25(OH)D_2_ and total 25(OH)D concentration were 59.8 (59.3–60.3), 3.3 (1.3–6.8) and 61.9 (49.9–74.9) nmol/L, respectively. The median (interquartile range) for serum PTH, phosphate and albumin-adjusted calcium were 4.5 (3.4–5.8) pmol/L, 1.53 (1.43–1.64) mmol/L and 2.37 (2.31–2.44) mmol/L, respectively.

Table 1 shows the distributions of SDQ scores in those children who were excluded from this study due to missing data and in those included. There were no differences in scores on any subscales, but complete cases had slightly lower total difficulty scores. Number (%) of children with borderline or abnormal behaviour were 86 (3.5%) for total difficulties, 171 (6.7%) for emotional symptoms, 255 (9.0%) for conduct problems 127 (4.9%) for hyperactivity 197 (8.1%) for peer problems and 101 (3.8%) for pro-social problems.

Table S1 shows the univariable associations of the potential confounders with the exposures. Higher BMI was associated with lower concentrations of 25(OH)D_3_ and 25(OH)D_2_ and higher concentrations of PTH. Non-white ethnicity was associated with higher PTH concentrations and children with higher IQ had lower albumin-adjusted calcium concentrations. Children from higher socioeconomic background had higher 25(OH)D_3_ concentrations and lower 25(OH)D_2_ and albumin-adjusted calcium concentrations. Children who spent more time outdoors during summer had higher 25(OH)D_3_ and 25(OH)D_2_ concentrations and those with higher UVB protection score had lower PTH concentrations. Family history of mental health problems was associated with lower 25(OH)D_3_ and albumin-adjusted calcium concentrations and higher PTH concentrations. Children with more advanced puberty stage had higher phosphate and albumin-adjusted calcium concentrations and lower 25(OH)D_3_ concentrations.

**Table 1:** Distribution of Strengths and Difficulties scores in children who were excluded due to missing data and in those included in at least one of the association analyses.

Outcome	Median (interquartile range), minimum, maximum, N	
	Excluded due to missing data	Included in at least one analysis
Total difficulties	5 (3, 9), min = 0 max = 34, N = 3636	5 (3, 8), min = 0 max = 27, N = 2561
Emotional symptoms	1 (0, 2), min = 0 max = 10, N = 4188	1 (0, 2), min = 0 max = 10, N = 2580
Conduct problems	1 (0, 2), min = 0 max = 10, N = 4151	1 (0, 2), min = 0 max = 9, N = 2841
Hyperactivity	2 (1, 4), min = 0 max = 10, N = 4144	2 (1, 4), min = 0 max = 10, N = 2838
Peer problems	0 (0, 2), min = 0 max = 9, N = 4008	0 (0, 2), min = 0 max = 9, N = 2755
Prosocial behaviour	9 (7, 10), min = 0 max = 10, N = 4184	9 (7, 10), min = 0 max = 10, N = 2840

Table 2 shows the univariable associations of confounders with prosocial problems and total difficulties at mean age 11.7 years. Higher BMI, lower IQ, lower socioeconomic position and more advanced puberty stage were associated with higher risk of behavioural problems. Children who had higher UVB protection score were less likely to have prosocial problems. Univariable associations with separate subscales of total difficulties were similar (Table S2).

**Table 2:** Univariable associations between potential confounders and prosocial problems and total difficulties.

	Prosocial problems		Total difficulties	
	Odds ratio per SD/category change (95%CI)	*P*	Odds ratio per SD/category change (95%CI)	*P*
BMI (kg/m^2^)	1.02 (0.98, 1.07)	0.27	1.02 (0.97, 1.07)	0.53
WISC full IQ score at 8.5 years^a^	0.95 (0.84, 1.07)	0.41	0.65 (0.57, 0.74)	<0.001
Non-white ethnicity	0.95 (0.84, 1.07)	0.12	1.14 (0.61, 2.12)	0.68
Head of household social class				
i	1 (reference)	0.87	1 (reference)	<0.001
ii	1.04 (0.73, 1.48)		1.15 (0.78, 1.72)	
iii non-manual	1.02 (0.69, 1.50)		1.56 (1.02, 2.36)	
iii manual	0.91 (0.55, 1.51)		1.39 (0.83, 2.35)	
iv/v	1.09 (0.56, 2.09)		2.83 (1.56, 5.13)	
Paternal education				
None/CSE	1 (reference)	0.10	1 (reference)	0.033
Vocational	0.99 (0.62, 1.58)		0.97 (0.59, 1.60)	
O level	0.91 (0.64, 1.30)		0.77 (0.52, 1.13)	
A level	0.72 (0.51, 1.02)		0.87 (0.61, 1.24)	
Degree	2.00 (1.13, 3.55)		0.62 (0.42, 0.92)	
Maternal education				
None/CSE	1 (reference)	0.025	1 (reference)	<0.001
Vocational	1.20 (0.76, 1.90)		0.81 (0.49, 1.31)	
O level	0.80 (0.56, 1.15)		0.59 (0.41, 0.85)	
A level	0.70 (0.47, 1.03)		0.54 (0.37, 0.80)	
Degree	0.75 (0.49, 1.15)		0.42 (0.27, 0.66)	
UVB protection score	0.90 (0.85, 0.95)	<0.001	0.97 (0.92, 1.03)	0.33
Average h/day spent outdoors during summer	1.05 (0.93, 1.18)	0.46	1.05 (0.93, 1.20)	0.43
Family history of depression/schizophrenia				
None	1 (reference)	0.77	1 (reference)	<0.001
Depression	1.10 (0.83, 1.46)		2.10 (1.58, 2.79)	
Schizophrenia	0.59 (0.14, 2.42)		1.91 (0.68, 5.35)	
Puberty stage at serum measurement				
1	1 (reference)	0.003	1 (reference)	0.008
2	1.50 (1.07, 2.10)		1.15 (0.79, 1.67)	
3	1.61 (1.10, 2.35)		1.72 (1.16, 2.56)	
4–5	1.87 (1.09, 3.19)		1.62 (0.89, 2.93)	

a IQ score was divided by 15 in order to produce odds ratios for a 15-point increase in IQ.

Table 3 shows the association of serum 25(OH)D_3_ and 25(OH)D_2_ concentrations with behavioural problems at mean age 11.7 years. Serum 25(OH)D_3_ concentrations were not associated with total difficulties, but when different subscales were studies, higher serum 25(OH)D_3_ concentrations were associated with lower risk of prosocial problems in the models adjusted for and potential confounders (Model 2) and serum concentrations of 25(OH)D_2_, phosphate, calcium and PTH (Model 3). Serum 25(OH)D_2_ concentrations were not associated with total difficulties. Higher serum 25(OH)D_2_ concentrations were weakly associated with higher risk of prosocial problems in the confounder-adjusted model (Model 2), but no longer after adjusting for other analytes. The associations of 25(OH)D_3_ and 25(OH)D_2_ with social problems were different (P = 0.005), but there was no strong statistical evidence that any of the associations of 25(OH)D_3_ with outcomes differed from those of 25(OH)D_2_ with the same outcomes (all other P for different effect ≥0.29). When participants who had 25(OH)D_3_ and 25(OH)D_2_ assessed on a blood sample that was taken at the same time as the outcome assessment (mean age 11.7) were excluded, the results were essentially the same as those including these participants (Table 4).

**Table 3:** Association of 25(OH)D_3_ and 25(OH)D_2_ concentrations with incident behavioural problems assessed by Strengths and Difficulties Questionnaire at mean age 11.7 years in children with exposures assessed at 7-,9- or 11-year clinic (mean age 9.8 years, N = 2413–2666^a^).

Exposure	Outcome^a^	Odds ratio for category change perdoubling of exposure (95%CI)		
		Model 1	Model 2	Model 3
25(OH)D_3_	Total difficulties	0.89 (0.77, 1.02)	0.88 (0.76, 1.02)	0.88 (0.75, 1.02)
	Emotional symptoms	0.96 (0.86, 1.06)	0.97 (0.87, 1.06)	0.97 (0.86, 1.08)
	Conduct problems	0.92 (0.82, 1.02)	0.92 (0.82, 1.03)	0.92 (0.82, 1.03)
	Hyperactivity	1.06 (0.94, 1.20)	1.05 (0.93, 1.17)	1.04 (0.91, 1.17)
	Peer relationship problems	0.94 (0.84, 1.04)	0.96 (0.87, 1.06)	0.95 (0.85, 1.05)
	Pro-social problems	0.84 (0.73, 0.96)	0.84 (0.72, 0.98)	0.85 (0.74, 0.98)
25(OH)D_2_	Total difficulties	0.88 (0.71, 1.09)	0.87 (0.69, 1.10)	0.85 (0.66, 1.06)
	Emotional symptoms	1.00 (0.84, 1.19)	0.99 (0.83, 1.21)	0.99 (0.81, 1.20)
	Conduct problems	1.02 (0.88, 1.18)	1.02 (0.88, 1.18)	1.02 (0.87, 1.20)
	Hyperactivity	1.06 (0.87, 1.28)	1.03 (0.83, 1.26)	1.03 (0.84, 1.25)
	Peer relationship problems	1.05 (0.90, 1.22)	1.05 (0.90, 1.22)	1.06 (0.90, 1.25)
	Pro-social problems	1.26 (0.97, 1.61)	1.30 (1.01, 1.68)	1.26 (0.97, 1.61)

Model 1 is unadjusted (the exposures are standardised for age and gender and 25(OH)D_3_ is adjusted for season and ethnicity).

Model 2 is adjusted for ethnicity, head of household social class, mothers and partners education, time spent outdoors during summer (age 8.5 years), UVB protection score, WISC IQ score at 8.5 years, BMI, family history of psychiatric problems and puberty stage.

Model 3 is adjusted for Model 2 plus serum concentrations of other hormones/metabolites which are related to vitamin D homoeostasis (eg. association of 25(OH)D_3_ is adjusted for 25(OH)D_2,_ phosphate, albumin-adjusted calcium and parathyroid hormone).

a The numbers included are the same for each model but differ by outcome: total difficulties n = 2413, emotional symptoms n = 2559, conduct problems n = 2502, hyperactivity n = 2570, peer problems n = 2447 and pro-social problems n = 2666.

**Table 4:** Association of 25(OH)D_3_ and 25(OH)D_2_ concentrations with incident behavioural problems assessed by Strengths and Difficulties Questionnaire at mean age 11.7 years in children with exposures assessed at 7- or 9-year clinic (mean age 9.4 years, N = 2072–2267^a^).

Exposure	Outcome^a^	Odds ratio for category change perdoubling of exposure (95%CI)		
		Model 1	Model 2	Model 3
25(OH)D_3_	Total difficulties	0.87 (0.74, 1.01)	0.87 (0.74, 1.01)	0.87 (0.73, 1.01)
	Emotional symptoms	0.96 (0.85, 1.06)	0.96 (0.86, 1.06)	0.96 (0.85, 1.07)
	Conduct problems	0.93 (0.83, 1.05)	0.93 (0.82, 1.05)	0.93 (0.82, 1.05)
	Hyperactivity	1.05 (0.93, 1.19)	1.05 (0.93, 1.19)	1.04 (0.90, 1.19)
	Peer relationship problems	0.95 (0.84, 1.05)	0.97 (0.86, 1.07)	0.96 (0.85, 1.08)
	Pro-social problems	0.84 (0.72, 0.97)	0.83 (0.70, 0.97)	0.83 (0.71, 0.97)
25(OH)D_2_	Total difficulties	0.90 (0.71, 1.14)	0.89 (0.69, 1.12)	0.87 (0.68, 1.10)
	Emotional symptoms	1.06 (0.87, 1.31)	1.06 (0.85, 1.31)	1.05 (0.84, 1.30)
	Conduct problems	1.02 (0.87, 1.20)	1.01 (0.85, 1.19)	1.00 (0.83, 1.18)
	Hyperactivity	1.12 (0.91, 1.35)	1.10 (0.88, 1.36)	1.10 (0.87, 1.37)
	Peer relationship problems	1.08 (0.90, 1.27)	1.07 (0.90, 1.26)	1.07 (0.90, 1.28)
	Pro-social problems	1.19 (0.90, 1.56)	1.22 (0.91, 1.63)	1.17 (0.87, 1.54)

Model 1 is unadjusted (the exposures are standardised for age and gender and 25(OH)D_3_ is adjusted for season and ethnicity).

Model 2 is adjusted for ethnicity, head of household social class, mothers and partners education, time spent outdoors during summer (age 8.5 years), UVB protection score, WISC IQ score at 8.5 years, BMI, family history of psychiatric problems and puberty stage.

Model 3 is adjusted for Model 2 plus serum concentrations of other hormones/metabolites which are related to vitamin D homoeostasis (eg. association of 25(OH)D_3_ is adjusted for 25(OH)D_2,_ phosphate, albumin-adjusted calcium and parathyroid hormone).

a numbers included are the same for each model but differ by outcome total difficulties n = 2072, emotional symptoms n = 2186, conduct problems n = 2139, hyperactivity n = 2197, peer problems n = 2097 and pro-social problems n = 2267.

Table S3 shows the association of serum albumin-adjusted calcium, phosphate and PTH concentrations with behavioural problems at mean age 11.7. Serum PTH concentrations were associated with higher risk of prosocial problems in the confounder-adjusted model (Model 2), but adjustment for other analytes attenuated this association towards the null. Phosphate or albumin-adjusted calcium concentrations were not associated with behavioural problems.

Table S4 shows the associations of unadjusted (for season) 25(OH)D_3_ and Table S5 shows the associations of unadjusted (for season) total 25(OH)D with behavioural problems at mean age 11.7 years. The associations were closer to the null value but otherwise similar to those shown in Table 3.

Table S6 shows the associations of season-adjusted25(OH)D_3_ and 25(OH)D_2_ with continuous SDQ scores at mean age 11.7 years. Neither 25(OH)D_3_ nor 25(OH)D_2_ were associated with total difficulties score or scores in any SDQ subscale.

## Discussion

Despite suggestive findings from animal studies, that vitamin D deficiency might be related to behavioural problems (reviewed in [16]–[18]), we found no strong or robust associations between serum 25(OH)D_3_ and 25(OH)D_2_ concentrations and behavioural problems in children. Only a weak association between lower 25(OH)D_3_ and higher 25(OH)D_2_ concentrations and higher risk of prosocial problems was observed. One previous small study (n = 206) in adults found that higher 1,25(OH)2D_3_ concentrations were associated with fewer behaviour problems [19], but as 1,25(OH)_2_D_3_ is the active hormone metabolite of vitamin D and its serum concentrations do not necessarily reflect vitamin D synthesis and intake [29], [30], which are more accurately reflected by serum 25(OH)D concentrations [1], [29], it is difficult to compare our results to those of the previous study [19]. Our findings are consistent with two previous studies that assessed the association between maternal 25(OH)D concentrations during pregnancy and behavioural problems in childhood and concluded with null results [20], [21]. Thus, vitamin D status during pregnancy or childhood does not seem to be related to behavioural problems in children.

Limitation of our study is small number of children who were classified as having behavioural problems on the basis of established cut-off values. However, the distribution of scores in each SDQ scale was similar to normative data from British 11–15 year-old children [31] and analysing the association with actual scores led to similar conclusions. Our sample was also considerably larger than in the previous studies assessing the association between vitamin D status in pregnancy and childhood behaviours (n = 177 in [21] and n = 743 in [20]).Other strengths of our study include prospective assessment of behavioural problems, exclusion children with previous behavioural problems to avoid reverse causality and the ability to examine potential confounding by a wide range of characteristics. We were also able to study the associations of 25(OH)D_3_ and 25(OH)D_2_ separately. The children who were lost to follow-up tended to be from lower socioeconomic background [25], but the distributions of SDQ scores were similar among those included and those who were excluded because of missing data on confounders or exposures. By including blood samples taken at the three clinics we minimised exclusion because of non-attendance at one of the follow-up clinics. The distribution of 25(OH)D concentrations was similar to other studies on Northern hemisphere [32], [33], so our results are likely to be generalisable to most Northern hemisphere populations with similar vitamin D concentrations, but may not generalise to those with very different dietary intakes or UVB exposure.

Although better vitamin D status, indicated by 25(OH)D concentrations has been associated with better cognitive function and mental health [2]–[4], [6]–[12], [34], higher serum concentrations of 25(OH)D_3_ and 25(OH)D_2_ were not associated with lower risk of behavioural problems. Thus, vitamin D status is unlikely to be a determinant of behavioural problems in children.

## Supporting Information

Table S1 Univariable associations between potential confounders and age and gender standardised serum 25-hydroxyvitamin D_3_, 25-hydroxyvitamin D_3,_ calcium, phosphate and PTH concentrations.
(DOC)

Table S2 Univariable associations between potential confounders and subscales of total difficulties (peer problems, conduct problems, emotional symptoms and hyperactivity).
(DOC)

Table S3 Association of phosphate, calcium and PTH concentrations with incident behavioural problems assessed by Strengths and Difficulties Questionnaire at mean age 11.7 (exposures assessed at 7-, 9- or 11-year clinics, mean age 9.8 years, N = 2413-2666^a^).
(DOC)

Table S4 Association of unadjusted (for season) 25(OH)D_3_ concentrations with incident behavioural problems assessed by Strengths and Difficulties Questionnaire at mean age 11.7 (exposures assessed at 7-, 9- or 11-year clinics, mean age 9.8 years, N = 2413-2666^a^).
(DOC)

Table S5 Association of total 25(OH)D concentrations with incident behavioural problems assessed by Strengths and Difficulties Questionnaire at mean age 11.7 (exposures assessed at 7-, 9- or 11-year clinics, mean age 9.8 years, N = 2413–2666^a^).
(DOC)

Table S6 Association of 25(OH)D_3_ and 25(OH)D_2_ concentrations with scores on total difficulties and subscales of Strengths and Difficulties Questionnaire at mean age 11.7 years in children with exposures assessed at 7-,9- or 11-year clinic (mean age 9.8 years, N = 2413–2666^a^) and in children with exposures assessed at 7- or 9-year clinic (mean age 9.4 years, N = 2072–2267^b^).
(DOC)
