# Supplementary material for: The Association of 25-Hydroxyvitamin D3 and D2 with Behavioural Problems in Childhood
Source: PLoS One. 2012 Jul 10;7(7):e40097. doi: 10.1371/journal.pone.0040097 (PMC3393748; doi:10.1371/journal.pone.0040097)
Supplement: Table S1 — Univariable associations between potential confounders and age and gender standardised serum 25-hydroxyvitamin D3, 25-hydroxyvitamin D3, calcium, phosphate and PTH concentrations. (DOC) [file pone.0040097.s001.doc]

Table S1. Univariable associations between potential confounders and age and gender standardised serum 25-hydroxyvitamin D_3_, 25-hydroxyvitamin D_3_, calcium, phosphate and PTH concentrations

|  | Season-adjusted 25(OH)D_3_ | | | | | 25(OH)D_2_ | | | Phosphate | | | Albumin-adjusted calcium | | | Parathyroid hormone | |
| --- | --- | --- | --- | --- | --- | --- | --- | --- | --- | --- | --- | --- | --- | --- | --- | --- |
|  | SD change per SD/category change (95%CI) | | | | P | SD change per SD/category change (95%CI) | P | | SD change per SD/category change (95%CI) | P | | SD change per SD/category change (95%CI) | | P | SD change per SD/category change (95%CI) | P |
| BMI (kg/m^2^) | -0.01  (-0.02, -0.01) | | | | 0.001 | -0.01  (-0.02, 0.00) | 0.01 | | 0.00  (-0.01, 0.01) | 0.81 | | 0.00  (-0.01, 0.01) | | 0.97 | 0.02  (0.01, 0.03) | <0.001 |
| WISC full IQ score at 8.5 years* | 0.01  (-0.02, 0.03) | | | | 0.60 | -0.01  (-0.02, 0.00) | 0.180 | | 0.01  (-0.01, 0.04) | 0.230 | | -0.03  (-0.05, -0.01) | | 0.010 | 0.00  (-0.03, 0.02) | 0.800 |
| Non-white ethnicity | N.A | | | |  | 0.00  (-0.12, 0.13) | 0.95 | | 0.01  (-0.10, 0.12) | 0.90 | | -0.01  (-0.12, 0.09) | | 0.81 | 0.34  (0.24, 0.45) | <0.001 |
| Head of household social class |  | | | |  |  |  | |  |  | |  | |  |  |  |
| i | -0.04  (-0.13, 0.04) | | | | 0.001 | 0.03  (-0.10, 0.17) | 0.012 | | -0.02  (-0.17, 0.13) | 0.07 | | 0.02  (-0.11, 0.15) | | 0.13 | 0.01  (-0.12, 0.14) | 0.54 |
| ii | 0.02  (-0.05, 0.09) | | | |  | 0.05  (-0.04, 0.13) |  | | -0.01  (-0.08, 0.06) |  | | 0.05  (-0.03, 0.12) | |  | 0.02  (-0.05, 0.09) |  |
| iii non-manual | -0.01  (-0.09, 0.07) | | | |  | 0.03  (-0.06, 0.12) |  | | -0.07  (-0.15, 0.00) |  | | 0.09  (0.01, 0.16) | |  | -0.04  (-0.12, 0.04) |  |
| iii manual | -0.14  (-0.23, -0.04) | | | |  | 0.18  (0.07, 0.29) |  | | -0.08  (-0.17, 0.02) |  | | 0.03  (-0.07, 0.13) | |  | 0.06  (-0.03, 0.16) |  |
| iv/v | -0.13  (-0.27, 0.00) | | | |  | 0.09  (-0.06, 0.24) |  | | -0.01  (-0.15, 0.12) |  | | 0.10  (-0.03, 0.23) | |  | 0.06  (-0.07, 0.19) |  |
| Paternal education |  | |  | | |  |  | |  |  | |  | |  |  |  |
| None/CSE | 0.02  (-0.11, 0.15) | | 0.03 | | | -0.03  (-0.10, 0.05) | 0.009 | | -0.03  (-0.10, 0.05) | 0.37 | | -0.03  (-0.10, 0.04) | | <0.001 | 0.00  (-0.08, 0.07) | 0.58 |
| Vocational | 0.11  (0.00, 0.21) |  | | | | -0.10  (-0.22, 0.02) |  | | 0.00  (-0.10, 0.10) |  | | -0.09  (-0.18, 0.01) |  | | -0.02  (-0.12, 0.08) |  |
| O level | 0.11  (0.03, 0.18) |  | | | | -0.01  (-0.09, 0.07) |  | | 0.01  (-0.07, 0.08) |  | | -0.06  (-0.14, 0.01) | |  | 0.02  (-0.05, 0.09) |  |
| A level | 0.13  (0.05, 0.20) |  | | | | -0.09  (-0.17, -0.01) | |  | -0.01  (-0.08, 0.06) |  | | -0.07  (-0.14, 0.00) | |  | 0.13  (0.05, 0.20) |  |
| Degree | 0.07  (0.00, 0.15) |  | | | | -0.12  (-0.20, -0.03) | |  | 0.05  (-0.03, 0.12) |  | | -0.15  (-0.22, -0.08) | |  | -0.02  (-0.10, 0.05) |  |
| Maternal education |  | |  | | |  |  | |  |  | |  | |  |  |  |
| None/CSE | 0.01  (-0.06, 0.09) | | 0.27 | | | -0.04  (-0.13, 0.05) | <0.001 | | 0.02  (-0.08, 0.11) | 0.12 | | -0.03  (-0.12, 0.05) | | <0.001 | 0.00  (-0.08, 0.09) | 0.76 |
| Vocational | 0.12  (0.01, 0.22) | | |  | | -0.12  (-0.23, 0.00) |  | | 0.09  (-0.02, 0.19) |  | | 0.02  (-0.09, 0.12) | |  | -0.11  (-0.22, -0.01) |  |
| O level | 0.13  (0.05, 0.20) | | |  | | -0.11  (-0.19, -0.02) | |  | -0.01  (-0.09, 0.06) |  | | -0.04  (-0.11, 0.04) | |  | -0.06  (-0.13, 0.01) |  |
| A level | 0.08  (0.00, 0.16) | | |  | | -0.15  (-0.24, -0.06) | |  | 0.03  (-0.05, 0.11) |  | | -0.07  (-0.14, 0.01) | |  | -0.03  (-0.10, 0.05) |  |
| Degree | 0.08  (-0.01, 0.17) | | |  | | -0.18  (-0.28, -0.09) | |  | 0.08  (0.00, 0.17) |  | | -0.14  (-0.23, -0.06) | |  | -0.02  (-0.11, 0.06) |  |
| UVB protection score | 0.00  (-0.01, 0.02) | | 0.62 | | | 0.00  (-0.01, 0.01) | 0.96 | | -0.01  (-0.02, 0.00) | 0.25 | | 0.01  (0.00, 0.02) | | 0.21 | -0.01  (-0.02, 0.00) | 0.07 |
| Average h/day spent outdoors during summer | 0.05  (0.02, 0.08) | | <0.001 | | | 0.02  (0.00, 0.05) | 0.052 | | 0.01  (-0.01, 0.04) | 0.28 | | 0.02  (-0.01, 0.04) | | 0.16 | -0.02  (-0.04, 0.00) | 0.12 |
| Family history of depression/schizophrenia |  | |  | | |  |  | |  |  | |  | |  |  |  |
| None | -0.09  (-0.19, 0.02) | | 0.002 | | | 0.06  (-0.16, 0.28) | 0.06 | | 0.00  (-0.25, 0.25) | 0.93 | | 0.01  (-0.20, 0.22) | | <0.001 | 0.01  (-0.20, 0.22) | <0.001 |
| Depression | -0.08  (-0.14, -0.02) | | | |  | 0.07  (0.01, 0.14) |  | | -0.01  (-0.07, 0.05) |  | | 0.03  (-0.03, 0.09) |  | | 0.02  (-0.04, 0.07) |  |
| Schizophrenia | -0.19  (-0.42, 0.03) | | |  | | 0.00  (-0.25, 0.25) |  | | 0.08  (-0.13, 0.29) |  | | -0.14  (-0.36, 0.07) |  | | -0.01  (-0.22, 0.20) |  |
| Puberty stage at serum measurement |  | |  | | |  |  | |  |  | |  | |  |  |  |
| 1 | -0.04  (-0.15, 0.07) | | 0.01 | | | 0.01  (-0.11, 0.13) | 0.56 | | 0.03  (-0.10, 0.15) | 0.07 | | 0.03  (-0.08, 0.13) | | 0.04 | 0.02  (-0.08, 0.13) | 0.08 |
| 2 | -0.06  (-0.12, 0.01) | | | |  | 0.03  (-0.04, 0.10) |  | | 0.04  (-0.02, 0.10) | |  | 0.03  (-0.03, 0.09) | |  | 0.04  (-0.01, 0.10) |  |
| 3 | -0.05  (-0.13, 0.02) | | | |  | -0.02  (-0.10, 0.06) |  | | 0.08  (0.01, 0.15) | |  | 0.02  (-0.05, 0.09) | |  | 0.09  (0.02, 0.16) |  |
| 4-5 | -0.14  (-0.26, -0.03) | | | |  | 0.08  (-0.04, 0.21) |  | | 0.03  (-0.08, 0.14) | |  | 0.14  (0.03, 0.24) | |  | 0.01  (-0.10, 0.11) |  |

*IQ score was divided by 15 in order to produce SD change in serum analyte per 15-point increase in IQ.
