# Supplementary material for: The Association of 25-Hydroxyvitamin D3 and D2 with Behavioural Problems in Childhood
Source: PLoS One. 2012 Jul 10;7(7):e40097. doi: 10.1371/journal.pone.0040097 (PMC3393748; doi:10.1371/journal.pone.0040097)
Supplement: Table S2 — Univariable associations between potential confounders and subscales of total difficulties (peer problems, conduct problems, emotional symptoms and hyperactivity). (DOC) [file pone.0040097.s002.doc]

Table S2. Univariable associations between potential confounders and subscales of total difficulties (peer problems, conduct problems, emotional symptoms and hyperactivity)

|  | Peer problems | | Conduct problems | | Emotional symptoms | | Hyperactivity | |
| --- | --- | --- | --- | --- | --- | --- | --- | --- |
|  | Odds ratio per SD/category change (95%CI) | P | Odds ratio per SD/category change (95%CI) | P | Odds ratio per SD/category change (95%CI) | P | Odds ratio per SD/category change (95%CI) | P |
| BMI (kg/m^2^) | 1.07 (1.04), 1.11) | <0.001 | 1.03 (1.00, 1.07) | 0.05 | 1.04 (1.00, 1.08) | 0.044 | 0.96 (0.92, 1.00) | 0.06 |
| WISC full IQ score at 8.5 years^a^ | 0.84 (0.76, 0.92) | <0.001 | 0.83 (0.76, 0.91) | <0.001 | 0.77 (0.70, 0.85) | <0.001 | 0.58 (0.51, 0.65) | <0.001 |
| Non-white ethnicity | 1.32 (0.87, 2.01) | 0.19 | 1.11 (0.72, 1.73) | 0.63 | 0.79 (0.46, 1.34) | 0.38 | 0.89 (0.50, 1.56) | 0.67 |
| Head of household social class | |  |  |  |  |  |  |  |
| i | 1 (reference) | <0.001 | 1 (reference) | 0.001 | 1 (reference) | 0.32 | 1 (reference) | 0.004 |
| ii | 1.37 (1.02, 1.84) |  | 1.20 (0.90, 1.59) |  | 1.25 (0.93, 1.68) |  | 1.47 (1.04, 2.08) |  |
| iii non-manual | 1.46 (1.07, 2.01) |  | 1.35 (1.00, 1.83) |  | 1.30 (0.95, 1.80) |  | 1.55 (1.06, 2.26) |  |
| iii manual | 1.90 (1.32, 2.74) |  | 1.53 (1.06, 2.21) |  | 1.18 (0.79, 1.76) |  | 1.84 (1.18, 2.87) |  |
| iv/v | 2.40 (1.51, 3.82) |  | 2.07 (1.31, 3.28) |  | 1.29 (0.76, 2.21) |  | 2.06 (1.16, 3.65) |  |
| Paternal education |  |  |  |  |  |  |  |  |
| None/CSE | 1 (reference) | 0.004 | 1 (reference) | <0.001 | 1 (reference) | 0.15 | 1 (reference) | <0.001 |
| Vocational | 0.70 (0.47, 1.02) |  | 0.96 (0.68, 1.37) |  | 1.14 (0.78, 1.67) |  | 0.98 (0.67, 1.45) |  |
| O level | 0.72 (0.55, 0.95) |  | 0.67 (0.51, 0.88) |  | 0.86 (0.63, 1.16) |  | 0.55 (0.40, 0.76) |  |
| A level | 0.76 (0.59, 0.97) |  | 0.72 (0.56, 0.93) |  | 0.89 (0.67, 1.17) |  | 0.62 (0.46, 0.83) |  |
| Degree | 0.63 (0.48, 0.83) |  | 0.58 (0.44, 0.77) |  | 0.84 (0.63, 1.14) |  | 0.47 (0.34, 0.65) |  |
| Maternal education |  |  |  |  |  |  |  |  |
| None/CSE | 1 (reference) | 0.007 | 1 (reference) | <0.001 | 1 (reference) | 0.012 | 1 (reference) | <0.001 |
| Vocational | 0.86 (0.59, 1.25) |  | 0.70 (0.48, 1.02) |  | 0.88 (0.59, 1.31) |  | 0.97 (0.63, 1.48) |  |
| O level | 0.69 (0.52, 0.90) |  | 0.70 (0.54, 0.92) |  | 0.78 (0.58, 1.04) |  | 0.78 (0.57, 1.07) |  |
| A level | 0.66 (0.49, 0.88) |  | 0.52 (0.39, 0.69) |  | 0.73 (0.54, 0.99) |  | 0.60 (0.42, 0.84) |  |
| Degree | 0.69 (0.50, 0.94) |  | 0.51 (0.37, 0.70) |  | 0.67 (0.47, 0.94) |  | 0.51 (0.34, 0.76) |  |
| UVB protection score | 0.99 (0.94, 1.03) | 0.50 | 0.96 (0.92, 1.00) | 0.07 | 0.96 (0.92, 1.01) | 0.11 | 0.99 (0.94, 1.04) | 0.61 |
| Average h/day spent outdoors during summer | 1.00 (0.91, 1.09) | 0.96 | 1.07 (0.97, 1.17) | 0.18 | 0.99 (0.90, 1.09) | 0.86 | 1.09 (0.97, 1.22) | 0.13 |
| Family history of depression/schizophrenia | | |  |  |  |  |  |  |
| None | 1 (reference) | <0.001 | 1 (reference) | 0.002 | 1 (reference) | <0.001 | 1 (reference) | <0.001 |
| Depression | 1.94 (1.59, 2.37) |  | 1.63 (1.33, 2.00) |  | 1.79 (1.44, 2.23) |  | 1.94 (1.53, 2.47) |  |
| Schizophrenia | 0.37 (0.09, 1.53) |  | 0.83 (0.33, 2.09) |  | 2.30 (1.16, 4.55) |  | 1.13 (0.41, 3.14) |  |
| Puberty stage at serum measurement | | |  |  |  |  |  |  |
| 1 | 1 (reference) | 0.43 | 1 (reference) | 0.001 | 1 (reference) | 0.56 | 1 (reference) | 0.019 |
| 2 | 1.31 (1.01, 1.68) |  | 1.21 (0.95, 1.56) |  | 0.75 (0.56, 0.99) |  | 1.08 (0.79, 1.47) |  |
| 3 | 1.04 (0.76, 1.43) |  | 1.61 (1.23, 2.11) |  | 0.95 (0.70, 1.30) |  | 1.55 (1.11, 2.16) |  |
| 4-5 | 1.09 (0.68, 1.76) |  | 1.53 (1.00, 2.34) |  | 1.13 (0.72, 1.77) |  | 1.36 (0.81, 2.28) |  |

^a^IQ score was divided by 15 in order to produce odds ratios for a 15-point increase in IQ.
