# Supplementary material for: The Association of 25-Hydroxyvitamin D3 and D2 with Behavioural Problems in Childhood
Source: PLoS One. 2012 Jul 10;7(7):e40097. doi: 10.1371/journal.pone.0040097 (PMC3393748; doi:10.1371/journal.pone.0040097)
Supplement: Table S3 — Association of phosphate, calcium and PTH concentrations with incident behavioural problems assessed by Strengths and Difficulties Questionnaire at mean age 11.7 (exposures assessed at 7-, 9- or 11-year clinics, mean age 9.8 years, N = 2413-2666a). (DOC) [file pone.0040097.s003.doc]

Table S3. Association of phosphate, calcium and PTH concentrations with incident behavioural problems assessed by Strengths and Difficulties Questionnaire at mean age 11.7 (exposures assessed at 7-, 9- or 11-year clinics, mean age 9.8 years, N=2413-2666^a^)

| Exposure | Outcome^a^ | OR for category change per doubling of exposure (95%CI) | | |
| --- | --- | --- | --- | --- |
|  |  | Model 1 | Model 2 | Model 3 |
| Albumin-adjusted calcium | Total difficulties | 1.09 (0.94, 1.26) | 1.07 (0.92, 1.23) | 1.10 (0.95, 1.26) |
|  | Emotional symptoms | 0.99 (0.89, 1.10) | 0.99 (0.88, 1.10) | 1.00 (0.90, 1.12) |
|  | Conduct problems | 1.01 (0.92, 1.11) | 0.99 (0.91, 1.09) | 0.99 (0.90, 1.10) |
|  | Hyperactivity | 1.07 (0.95, 1.20) | 1.04 (0.93, 1.17) | 1.05 (0.92, 1.19) |
|  | Peer relationship problems | 1.02 (0.92, 1.12) | 1.02 (0.92, 1.13) | 1.00 (0.90, 1.10) |
|  | Pro-social problems | 1.04 (0.91, 1.20) | 1.04 (0.91, 1.19) | 1.05 (0.91, 1.20) |
| Phosphate | Total difficulties | 0.94 (0.79, 1.13) | 0.95 (0.82, 1.17) | 0.93 (0.81, 1.11) |
|  | Emotional symptoms | 0.94 (0.84, 1.06) | 0.95 (0.86, 1.06) | 0.94 (0.84, 1.06) |
|  | Conduct problems | 0.99 (0.90, 1.09) | 1.00 (0.91, 1.11) | 1.00 (0.90, 1.11) |
|  | Hyperactivity | 0.95 (0.84, 1.12) | 0.94 (0.82, 1.11) | 0.95 (0.81, 1.15) |
|  | Peer relationship problems | 1.05 (0.95, 1.16) | 1.06 (0.95, 1.17) | 1.07 (0.96, 1.19) |
|  | Pro-social problems | 1.00 (0.89, 1.12) | 0.99 (0.88, 1.12) | 0.95 (0.85, 1.07) |
| Parathyroid hormone | Total difficulties | 1.06 (0.90, 1.22) | 1.06 (0.89, 1.27) | 1.07 (0.90, 1.28) |
|  | Emotional symptoms | 1.06 (0.95, 1.18) | 1.06 (0.95, 1.19) | 1.07 (0.95, 1.21) |
|  | Conduct problems | 1.00 (0.91, 1.10) | 1.00 (0.91, 1.10) | 0.99 (0.90, 1.09) |
|  | Hyperactivity | 0.94 (0.83, 1.07) | 0.94 (0.83, 1.06) | 0.95 (0.84, 1.08) |
|  | Peer relationship problems | 0.96 (0.86, 1.05) | 0.95 (0.86, 1.05) | 0.93 (0.83, 1.03) |
|  | Pro-social problems | 0.95 (0.86, 1.05) | 1.15 (1.01, 1.33) | 1.12 (0.97, 1.31) |

Model 1 is unadjusted (the exposures are standardised for age and gender and 25(OH)D_3_ is adjusted for season and ethnicity)

Model 2 is adjusted for ethnicity, head of household social class, mothers and partners education, time spent outdoors during summer (age 8.5 years), UVB protection score, WISC IQ score at 8.5 years, BMI, family history of psychiatric problems and puberty stage

Model 3 is adjusted for Model 2 plus serum concentrations of other hormones/metabolites which are related to vitamin D homoeostasis (eg. association of phosphate is adjusted for 25(OH)D_3_, 25(OH)D_2_, albumin-adjusted calcium and parathyroid hormone)

^a^The numbers included are the same for each model but differ by outcome: total difficulties n=2413, emotional symptoms n=2559, conduct problems n=2502, hyperactivity n=2570, peer problems n=2447 and pro-social problems n=2666
