# Supplementary material for: The Association of 25-Hydroxyvitamin D3 and D2 with Behavioural Problems in Childhood
Source: PLoS One. 2012 Jul 10;7(7):e40097. doi: 10.1371/journal.pone.0040097 (PMC3393748; doi:10.1371/journal.pone.0040097)
Supplement: Table S5 — Association of total 25(OH)D concentrations with incident behavioural problems assessed by Strengths and Difficulties Questionnaire at mean age 11.7 (exposures assessed at 7-, 9- or 11-year clinics, mean age 9.8 years, N = 2413–2666a). (DOC) [file pone.0040097.s005.doc]

Table S5. Association of total 25(OH)D concentrations with incident behavioural problems assessed by Strengths and Difficulties Questionnaire at mean age 11.7 (exposures assessed at 7-, 9- or 11-year clinics, mean age 9.8 years, N=2413-2666^a^)

| Outcome* | OR for category change per doubling of exposure (95%CI) | | |
| --- | --- | --- | --- |
|  | Model 1 | Model 2 | Model 3 |
| Total difficulties | 0.95 (0.82, 1.12) | 0.94 (0.81, 1.10) | 0.96 (0.82, 1.13) |
| Emotional symptoms | 1.01 (0.91, 1.12) | 0.99 (0.90, 1.10) | 1.01 (0.90, 1.12) |
| Conduct problems | 0.96 (0.88, 1.06) | 0.97 (0.88, 1.06) | 0.96 (0.88, 1.07) |
| Hyperactivity | 1.01 (0.89, 1.14) | 0.98 (0.87, 1.11) | 0.98 (0.87, 1.12) |
| Peer relationship problems | 1.01 (0.90, 1.13) | 1.03 (0.92, 1.15) | 1.01 (0.90, 1.14) |
| Pro-social problems | 0.92 (0.80, 1.05) | 0.91 (0.79, 1.04) | 0.93 (0.81, 1.09) |

Model 1 is unadjusted (the exposures are standardised for age and gender)

Model 2 is adjusted for ethnicity, head of household social class, mothers and partners education, time spent outdoors during summer (age 8.5 years), UVB protection score, WISC IQ score at 8.5 years, BMI, family history of psychiatric problems and puberty stage

Model 3 is adjusted for Model 2 plus serum concentrations of albumin-adjusted calcium, phosphate and parathyroid hormone)

^a^The numbers included are the same for each model but differ by outcome: total difficulties n=2413, emotional symptoms n=2559, conduct problems n=2502, hyperactivity n=2570, peer problems n=2447 and pro-social problems n=2666
