# Supplementary material for: The Association of 25-Hydroxyvitamin D3 and D2 with Behavioural Problems in Childhood
Source: PLoS One. 2012 Jul 10;7(7):e40097. doi: 10.1371/journal.pone.0040097 (PMC3393748; doi:10.1371/journal.pone.0040097)
Supplement: Table S6 — Association of 25(OH)D3 and 25(OH)D2 concentrations with scores on total difficulties and subscales of Strengths and Difficulties Questionnaire at mean age 11.7 years in children with exposures assessed at 7-,9- or 11-year clinic (mean age 9.8 years, N = 2413–2666a) and in children with exposures assessed at 7- or 9-year clinic (mean age 9.4 years, N = 2072–2267b). (DOC) [file pone.0040097.s006.doc]

Table S6. Association of 25(OH)D_3_ and 25(OH)D_2_ concentrations with scores on total difficulties and subscales of Strengths and Difficulties Questionnaire at mean age 11.7 years in children with exposures assessed at 7-,9- or 11-year clinic (mean age 9.8 years, N=2413-2666^a^) and in children with exposures assessed at 7- or 9-year clinic (mean age 9.4 years, N=2072-2267^b^)

| Exposure | Outcome | Odds ratio for category change per doubling of exposure (95%CI) | | |
| --- | --- | --- | --- | --- |
|  |  | Model 1 | Model 2 | Model 3 |
| Exposures assessed at 7-, 9- or 11-year clinic^a^ | |  |  |  |
| 25(OH)D_3_ | Total difficulties | 0.99 (0.97, 1.01) | 0.99 (0.98, 1.01) | 0.99 (0.98, 1.01) |
|  | Emotional symptoms | 0.99 (0.96, 1.02) | 1.00 (0.96, 1.02) | 0.99 (0.96, 1.02) |
|  | Conduct problems | 0.99 (0.96, 1.02) | 0.99 (0.96, 1.02) | 0.99 (0.96, 1.02) |
|  | Hyperactivity | 1.00 (0.98, 1.02) | 1.00 (0.98, 1.02) | 0.97 (0.94, 1.01) |
|  | Peer relationship problems | 0.98 (0.95, 1.02) | 0.98 (0.95, 1.02) | 1.00 (0.98, 1.02) |
|  | Pro-social problems | 0.99 (0.96, 1.01) | 0.99 (0.96, 1.01) | 0.99 (0.97, 1.01) |
| 25(OH)D_2_ | Total difficulties | 1.01 (0.98, 1.04) | 1.01 (0.98, 1.04) | 1.01 (0.98, 1.04) |
|  | Emotional symptoms | 0.99 (0.95, 1.03) | 0.99 (0.94, 1.03) | 0.98 (0.94, 1.03) |
|  | Conduct problems | 1.02 (0.98, 1.07) | 1.02 (0.98, 1.07) | 1.02 (0.97, 1.07) |
|  | Hyperactivity | 1.02 (0.98, 1.05) | 1.01 (0.98, 1.04) | 1.01 (0.98, 1.04) |
|  | Peer relationship problems | 1.02 (0.97, 1.08) | 1.03 (0.98, 1.09) | 1.03 (0.98, 1.09) |
|  | Pro-social problems | 1.01 (0.97, 1.05) | 1.01 (0.98, 1.06) | 1.01 (0.97, 1.05) |
|  |  |  |  |  |
| Exposures assessed at 7- or 9-year clinic^b^ | |  |  |  |
| 25(OH)D_3_ | Total difficulties | 0.99 (0.97, 1.01) | 0.99 (0.97, 1.01) | 0.99 (0.97, 1.01) |
|  | Emotional symptoms | 0.99 (0.96, 1.03) | 1.00 (0.97, 1.03) | 1.00 (0.96, 1.03) |
|  | Conduct problems | 0.99 (0.96, 1.02) | 1.00 (0.97, 1.03) | 1.00 (0.96, 1.03) |
|  | Hyperactivity | 1.00 (0.98, 1.02) | 1.00 (0.98, 1.02) | 1.00 (0.98, 1.02) |
|  | Peer relationship problems | 0.97 (0.94, 1.01) | 0.98 (0.95, 1.02) | 0.99 (0.95, 1.03) |
|  | Pro-social problems | 0.99 (0.96, 1.02) | 0.99 (0.96, 1.02) | 0.99 (0.96, 1.02) |
| 25(OH)D_2_ | Total difficulties | 1.01 (0.98, 1.05) | 1.01 (0.98, 1.05) | 1.01 (0.98, 1.04) |
|  | Emotional symptoms | 1.01 (0.96, 1.07) | 1.01 (0.96, 1.06) | 1.00 (0.95, 1.06) |
|  | Conduct problems | 1.02 (0.97, 1.08) | 1.02 (0.97, 1.07) | 1.02 (0.97, 1.07) |
|  | Hyperactivity | 1.01 (0.98, 1.05) | 1.01 (0.97, 1.05) | 1.01 (0.97, 1.04) |
|  | Peer relationship problems | 1.02 (0.97, 1.09) | 1.04 (0.97, 1.10) | 1.03 (0.97, 1.10) |
|  | Pro-social problems | 1.01 (0.96, 1.05) | 1.00 (0.96, 1.05) | 1.00 (0.95, 1.05) |

Model 1 is unadjusted (the exposures are standardised for age and gender and 25(OH)D_3_ is adjusted for season and ethnicity)

Model 2 is adjusted for ethnicity, head of household social class, mothers and partners education, time spent outdoors during summer (age 8.5 years), UVB protection score, WISC IQ score at 8.5 years, BMI, family history of psychiatric problems and puberty stage

Model 3 is adjusted for Model 2 plus serum concentrations of other hormones/metabolites which are related to vitamin D homoeostasis (eg. association of 25(OH)D_3_ is adjusted for 25(OH)D_2_, phosphate, albumin-adjusted calcium and parathyroid hormone)

^a^The numbers included are the same for each model but differ by outcome: total difficulties n=2413, emotional symptoms n=2559, conduct problems n=2502, hyperactivity n=2570, peer problems n=2447 and pro-social problems n=2666

^b^The numbers included are the same for each model but differ by outcome total difficulties n=2072, emotional symptoms n=2186, conduct problems n=2139, hyperactivity n=2197, peer problems n=2097 and pro-social problems n=2267
